# High-Temperature Requirement A1 (Htra1) - A Novel Regulator of Canonical Wnt Signaling

**DOI:** 10.1038/s41598-017-18203-2

**Published:** 2017-12-21

**Authors:** Oriane Globus, Tamar Evron, Michal Caspi, Ronen Siman-Tov, Rina Rosin-Arbesfeld

**Affiliations:** Department of Clinical Microbiology and Immunology, Sackler School of Medicine, Tel Aviv University, Tel Aviv, 69978 Israel

## Abstract

Different cancer types as well as many other diseases are caused by aberrant activation of the canonical Wnt signal transduction pathway, and it is especially implicated in the development and progression of colorectal cancer (CRC). The main effector protein of the canonical Wnt signaling cascade is β-catenin, which binds to the T- cell factor/lymphoid enhancer factor (TCF/LEF) and triggers the activation of Wnt target genes. Here, we identify the serine protease High-Temperature Requirement A1 (HTRA1) as a novel component of the canonical Wnt pathway. We show that the HTRA1 protein inhibits the Wnt/β-catenin signaling, in both paracrine and autocrine manners, and affects the expression of several Wnt target genes. Moreover, HTRA1 forms a complex with β-catenin and reduces the proliferation rates of cells. Taken together, our findings indicate that HTRA1 functions as a novel suppressor of the canonical Wnt signaling pathway.

## Introduction

The canonical Wnt signaling pathway, also called the Wnt/β-catenin signaling pathway, is transduced by the stabilization of β-catenin following the interaction between a specific Wnt ligand and its designated receptors. This important developmental pathway mainly controls embryonic growth but is also implicated in adult tissue maintenance. Thus, abnormal constitutive stimulation of the canonical Wnt cascade leads to irregular cell proliferation and promotes the progression of numerous types of human cancers, particularly the human colorectal cancers (CRCs)^1–4^. The expression levels of cytosolic β-catenin, the Wnt signaling pathway’s key effector, are tightly regulated by the ‘β-catenin destruction complex’, which is usually active in the absence of a Wnt signal. This protein complex encompasses large number of components including the tumor suppressor protein adenomatous polyposis coli (APC) and Axin, as well as the kinases glycogen synthase kinase (GSK3β) and casein kinase-1 (CK1), which phosphorylate β-catenin, marking it for ubiquitination following by proteasomal degradation^5–7^. Mutations in APC, Axin, or β-catenin disrupt this ‘degradation complex’, resulting in the accumulation of β-catenin and an aberrant activation of Wnt target genes. Initiation of the Wnt pathway is triggered by Wnt ligand binding to the Frizzled (Fz) transmembrane receptor and the co-receptor low-density lipoprotein receptor-related 5 (LRP5) or LRP6^8,9^. This complex induces the association of Axin or the entire ‘destruction complex’ with LRP, which leads to a cascade of events that include Disheveled (Dvl) polymerization, which promotes the accumulation of unphosphorylated β-catenin that translocates into the nucleolus^4^. In the nucleus, β-catenin forms a complex with the TCF/LEF transcription factors and induces the upregulation of Wnt target genes. Indeed, aberrant expression of Wnt target genes is highly implicated in many cases of neoplastic transformation^10,11^. Wnt signaling is extremely intricate, regulated by other cascades and feedback loops and comprises numerous components, yet to be identified. We have successfully utilized a mammalian cells screening technique^12^ in order to isolate new Wnt signaling co  mponents. One of the genes identified was High-Temperature Requirement A1 (HTRA1). HTRA1 is a conserved PDZ serine protease, a member of the HTRA family of serine proteases, which is involved in numerous basal biological mechanisms in mammals^13^. It is a secreted enzyme with a widespread pattern of expression, and its levels in human tissues are modulated by different physiological activities^14^. HTRA1 is composed of four distinct protein domains: an Insulin-like growth factor binding domain (IGF-BD), a kazal-type motif (KM), a trypsin-like peptidase (proteolytic) domain, and a PDZ domain. The signal peptide (SP) in the N-terminus of the protein is essential for both the expression and the secretion of the HTRA1 protein. Human HTRA1 has been implicated in several severe pathologies such as the cerebral small vessel and arthritic diseases as well as in age-related macular degeneration^15–17^. This suggests that HTRA1 plays an important role in human physiology. In addition, several publications link the HTRA1 gene to tumorigenesis, since it has been found to be down-regulated in many tumors such as prostate cancer, medulloblastoma^18^, ovarian cancer^19^, melanoma^20^, lung carcinoma^21^, and mesothelioma^22^. In breast cancer, several studies indicate that HTRA1 expression is lower in estrogen-receptor(ER)-negative tumors and that down-regulation of HTRA1 is significantly correlated with a higher grade of breast carcinoma^23^. Moreover, poorly differentiated breast tumors and those with mutant p53 or with lymphatic infiltration have significantly lower levels of HTRA1 expression^24^. Recently it has been shown that epigenetic silencing of the HTRA1 gene was linked to numerous cancerous phenotypes, and specifically to colorectal cancer. Importantly, it was shown that the HTRA1 promoter was methylated and repressed in a mouse model of the human familial adenomatous polyposis disease^25^.

In the present study, we show that in accordance with its tumor suppressor traits, HTRA1 reduces the oncogenic Wnt signal, the β-catenin expression levels, and the proliferative abilities of cells. Additionally, we show that HTRA1 interacts with β-catenin and that its effect on the Wnt signal is mediated through β-catenin degradation by the proteasome. These findings indicate that HTRA1 may be a novel regulator involved in controlling the canonical Wnt cascade.

## Materials and Methods

### Cell culture, transfection

HEK293T, L-Wnt3a, L, COS-7, and the human CRC SW480 and HCT-116 cell lines were maintained in DMEM supplemented with 10% FCS as previously described^12^. HEK293T cells were tranfected by CaCl_2_ precipitation. Transfections of all other cells performed with jetPEI (Polyplus Transfection, Illkirch, France) following the manufacturer’s protocols.

### Plasmids and reagents

HTRA1 constructs: the HTRA1-GFP-N3 plasmid was constructed by amplifying the genomic cDNA by PCR and subcloning it into enhanced (pEGFP)-N3 vector (Clontech), using the XhoI and BamHI restriction sites. The HTRA1-HA plasmid was constructed by amplifying the genomic cDNA by PCR and subcloning it into an enhanced pcDNA3.1/N-HA vector, using the XhoI and BamHI restriction sites. The HTRA1-HA and HTRA1-GFP expression vectors were used as templates for the mutagenesis of HTRA1; serine 328 was replaced with alanine (designated HTRA1-HA-S328A and HTRA1-GFP-S328A). GFP-Kazal, GFP-IGFBD, GFP-Protease, and GFP-PDZ were cloned by a PCR reaction using full-length HTRA1-pEGFP-N3 as a template, and the amplicons were inserted into the pEGFP-C2 vector (Clontech, Oxon, UK) using the Gibson Chew Back and Anneal Assembly, according to the manufacturer’s protocol. All fragments containing the domains’ cDNAs were extracted from agarose gel using the DNA cleaning kit (Wizard® SV Gel and PCR clean-Up System, Promega) according to the manufacturer’s instructions. All β-catenin and luciferase system constructs were obtained as previously described^12^. MG132 (Calbiochem, San Diego, CA, USA) and TGF-β (H8541-5UG; Sigma, Israel) were used as indicated.

### Luciferase reporter, Western blot and immunoprecipitation (IP) assays

All these experiments were conducted as previously described^12^.

### Immunofluorescence

COS-7, L-Wnt3a, SW480 or HCT-116 cells were grown and stained as described previously^12^.

### Antibodies

The antibodies that were used include: mouse anti-β-catenin (IB: 1:5000; IF: 1:300; BD Transduction Laboratories, Franklin Lakes, NJ, USA), mouse anti-active β-catenin (IB: 1:1000; IF: 1:150; Anti-ABC clone 8E7; Merck Millipore, Temecula, CA, USA), rabbit anti-GFP (1:1000; Santa Cruz Biotechnology), rat anti-HA (IB: 1:2500; IF: 1:300; Roche, Indianapolis, IN, USA), mouse anti-FLAG (1:5000; Sigma), mouse anti-HTRA1 (IB: 1:500; IF: 1:50; IHC 1:50; R&D Systems, Minneapolis, MN, USA), rabbit anti-HTRA1 (IF: 1:50; IB 1:500; PRSS11 (C-term), Acris Antibodies GmbH), rabbit anti-HTRA1 (IF: 1:50; IB 1:1000; Abgent, Inc.) rabbit anti-Golgin-97 (IF: 1:100; (D8P2K) Cell Signaling Technology, Inc.). Mouse anti-tubulin (1:10000; Sigma) was used as a loading control. Anti-rat horseradish peroxidase-conjugated secondary antibodies were obtained from Santa Cruz Biotechnology (1:5000). Anti-mouse and anti-rabbit secondary antibodies were obtained from Jackson Immuno Research (West Grove, PA, USA) (1:10000). For IF, Alexa red and green (1:500; Molecular Probes, Grand Island, NY, USA) were used.

### Small interfering assay

HTRA1 small interfering RNA (siRNA) oligonucleotides were purchased from Thermo Scientific Dharmacon (Chicago, IL, USA; siGENOME SMARTpool, siGENOME Human HTRA1 siRNA; NM_002775; M-006009-02-0005). The transfection reagent Interferine was purchased from Polyplus-France. Transfection was performed according to the manufacturer’s protocol. All experiments were performed in HEK293T cells using 100 nM siRNA oligonucleotides for 72 h. Non-targeting RNA oligonucleotides (Thermo Scientific Dharmacon) were used as a control. HEK293T cells were transfected 24 h after siRNA transfection with pTOPFLASH/ pFOPFLASH and β-gal plasmids to detect luciferase. Forty-eight hours post-transfection, the cells were harvested and subjected to either luciferase assay or Western blot analysis as described above. Alternatively, 72 hours after HTRA1 siRNA transfection, the cells were harvested; total RNA was extracted from the cells and converted into more stable cDNA. Then, cDNA from the cells was subjected to qRT- PCR assay.

### Conditioned Media (CM)

HEK293T cells were transfected with HTRA1GFP or GFP vectors and the media was collected 24–48 h later and used as HTRA1 cultured medium.

### Proliferation assay

Cell proliferation was measured using an XTT cell proliferation kit (Sigma-Aldrich, cat. No. 11465015001). Cells were grown in a 24-well plate. Twenty-four hours following transfection, cells were split into a 96-well plate. Following transfection (48 h later) the cells were treated with a XTT labeling mixture according to the manufacturer’s instructions. Cells were incubated at 37 °C, and the proliferation levels were measured every 4 h, 18 h, and 24 h.

### RNA extraction and cDNA synthesis

Total RNA was isolated from the cultured cells according to the protocol supplied with TRI Reagent (Sigma-Aldrich). The concentration and purity of the RNA samples were determined and total RNA was reverse transcribed (RT) with the iScript cDNA Synthesis Kit (BioRad) according to the manufacturer’s instructions.

### Real-time PCR

All real-time PCR reactions were performed using the CFX Connect Real-Time PCR Detection System (BioRad) and the amplifications were carried out using Fast Start Universal SYBR Green Master (Roche). The thermal cycling conditions were as follows: 50 °C for 2 min, followed by an initial denaturation step at 95 °C for 10 min, 40 cycles at 95 °C for 15 s, and 60 °C for 60 s. The experiments were carried out in three independent experiments in triplicates. β-actin was used as an endogenous control to normalize each sample.

The primers for the amplifications of the specific cDNA sequences were as follows:

HTRA1 Fw: 5′AAAGCCAAAGAGCTGAAGGACC3′

HTRA1 Rv: 5′ACATCATTGGCGGAGACCAC3′

Axin2 Fw: 5′GCAGCTCAGCAAAAAGGGAAAT3′

Axin2 Rv: 5′TACATCGGGAGCACCGTCTCAT3′

C-myc Fw: 5′CTCGGTGGTCTTCCCCTACCCT3′

C-myc Rv: 5′TGTCCAACTTGACCCTCTTGGC3′

Cyclin D Fw: 5′CATCTACACCGACAACTCCATC3′

Cyclin D Rv: 5′TCTGGCATTTTGGAGAGGAAG3′

β-actin Fw: 5′CCTGGCACCCAGCACAAT3′

β-actin Rv: 5′GGGCCGGACTCGTCATACT3′

### Serine protease inhibitor assay

HEK293T cells were grown in a 6-well plate and transfected with the indicated plasmids. Following transfection, cells were incubated with 50 μM serine protease inhibitor 4-(2-Aminoethyl) benzenesulfonyl fluoride hydrochloride (AEBSF) (Sigma-Aldrich, CAS no. 30827-99-7) for 30 min at 37 °C and then harvested.

## Results

### HTRA1 affects Wnt signaling

**Figure 1:**
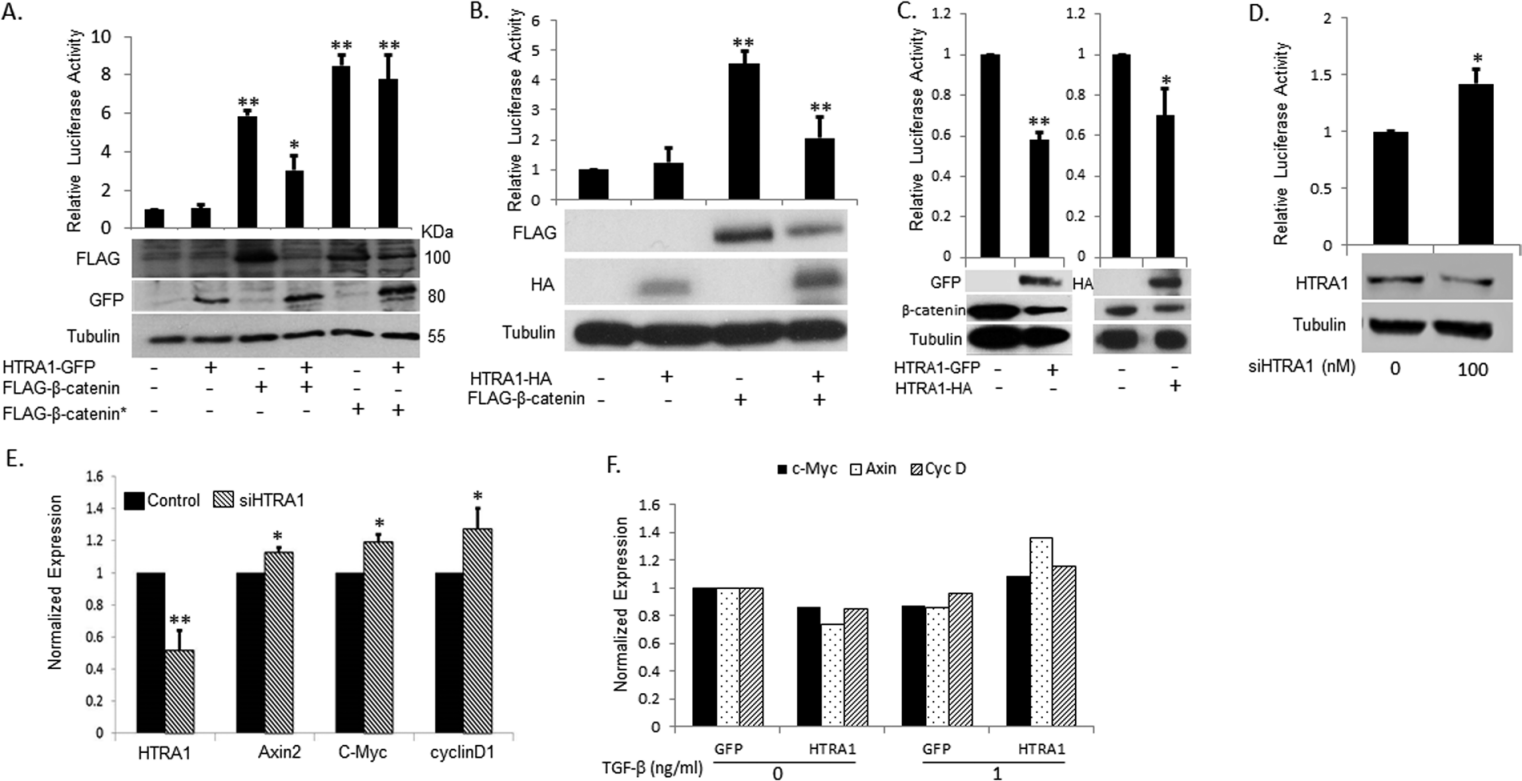
HTRA1 decreases the canonical Wnt Signal. (**A**) HEK293T cells were transfected with the pTOPFLASH and pFOPFLASH constructs (0.5 μg) and β-gal plasmid (0.1 μg), along with HTRA1-GFP, FLAG-β-catenin, FLAG-β-catenin-mut, or an empty pEGFP vector as a basal control (1 μg). Following transfection (48 h), cells were harvested and subjected to a luciferase assay. The results depict the relative luciferase values (pTOPFLASH/pFOPFLASH) normalized by β-gal expression. Each bar represents the mean ± SD of three independent experiments (performed in triplicate). Cells were harvested and the lysates were subjected to Western blot analysis, using anti-FLAG, anti-GFP, or anti-tubulin antibodies and subsequently with anti-mouse HRP or anti-rabbit HRP antibodies. Tubulin served as a loading control. (**B**) HEK293T cells were transfected as in A along with HTRA1-HA, FLAG-β-catenin, or an empty HA vector as a basal control (1 μg). Forty-eight hours after transfection, cells were harvested and subjected to a luciferase assay. Western blot analysis was conducted as in A using anti-FLAG or anti-tubulin antibodies. (**C**) SW480 cells were co-transfected with HTRA1-GFP or an empty pEGFP vector as a basal control (1 μg), pTOPFLASH or pFOPFLASH (0.5 μg), and renilla (0.1 μg). Alternatively, cells were co-transfected with HTRA1-HA or an empty HA vector as basal control (1 μg), pTOPFLASH or pFOPFLASH (0.5 μg) and β-gal (0.1 μg). Forty-eight hours later, cells were harvested and lysates were measured for their luciferase activity and then separated by SDS-PAGE. Blots were incubated with anti-GFP, anti-HA, anti-β-catenin, or anti-tubulin antibodies. (**D**) HEK293T cells were transfected with HTRA1 siRNA (100 nM) or scrambled siRNA as a control. Seventy two hr later cells were harvested and the lysates were subjected to Western blot analysis using anti-HTRA1 or anti-tubulin antibodies. HEK293T cells were transfected with HTRA1 siRNA (100 nM). Twenty-four hours later, cells were transfected with pTOPFLASH/pFOPFLASH constructs (0.5 μg) and β-gal plasmid (0.1 μg). Forty-eight hours after transfection, the cells were harvested and subjected to a luciferase assay as before. (**E**) HEK293T cells were transfected with HTRA1 siRNA (100 nM) or scrambled siRNA as a control. Seventy-two hours after transfection, cells were harvested; total RNA was extracted from the cells and converted into a more stable cDNA. Then, cDNA from the cells was subjected to real-time PCR assay. The results shown demonstrate the relative starting quantity for the HTRA1 gene and the Wnt target genes Axin-2, c-myc, and Cyclin D1, normalized by actin expression. Each bar represents the mean ± SD of three independent experiments. (*p value < 0.05 **p value < 0.01, Student’s t-test). (**F**) SW480 cells were transfected with GFP or HtRA1-GFP. Twenty four hours later, TGF-β was added for an additional 20 houres and RNA was extracted and converted into cDNA. The cDNA was subjected to quantitative real-time PCR assay. The results shown demonstrate the relative starting quantity for the Wnt target genes Axin-2, c-Myc, and Cyclin D1, normalized by actin expression.

One of the genes isolated in our screen, aimed at identifying new Wnt signaling regulators, was HTRA1^12^. Since most of the cDNAs used for this screen enclosed only the candidate’s C-terminal parts, the full-length HTRA1 cDNA was isolated and sub-cloned into GFP- or HA-tagged expression vectors. The HTRA1 expressing plasmids were first examined for their capacity to affect Wnt signaling in a specific manner by measuring the β-catenin/TCF-mediated transcription (using the pTOPFLASH/pFOPFLASH reporter assay). As shown in Fig. 1A,B, overexpression of HTRA1 in HEK293T cells expressing β-catenin resulted in a reduced signal. Importantly, a complete reduction in the levels of wild type β-catenin was detected in the presence of HTRA1. Interestingly, the decrease in Wnt signaling was abolished when the mutated form of β-catenin (that cannot be phosphorylated and thus is protected from proteasomal degradation) was used to activate the Wnt signal. Similar results were shown when the Wnt signal was activated with Wnt-3a CM (not shown). Moreover, HTRA1 reduced the Wnt signal in the colorectal cell line SW480 where the Wnt signaling pathway is constitutively active due to a mutation in the APC gene, which inactivates the β-catenin destruction complex (Fig. 1C). This may suggest that β-catenin phosphorylation, but not the integrity of the degradation complex, is crucial for the involvement of HTRA1. Next, HTRA1 silencing in HEK293T cells was performed using an RNA smart pool. As expected, HTRA1 depletion led to the activation of Wnt signaling in cells, as can be seen in Fig. 1D. In addition, several Wnt target genes, namely, axin2, C-myc, and cyclinD1, were significantly upregulated following HTRA1 silencing in comparison with the control RNA (Fig. 1E). Taken together, these results indicate that HTRA1 acts as an inhibitor of the canonical Wnt signaling pathway. As several studies have shown that the Wnt cascade and TGF-β signaling crosstalk and that HTRA1 may function through TGF-β, we examined the effect of TGF-β on HTRA1-affected Wnt target genes. Indeed, our results (Fig. 1F) indicate that the addition of TGF-β to SW480 cells overexpressing HTRA1-GFP interferes with the effect of HTRA1 on the Wnt target genes: c-myc, axin-2, and cyclinD1.

### Secreted HTRA1 affects canonical Wnt signaling

**Figure 2:**
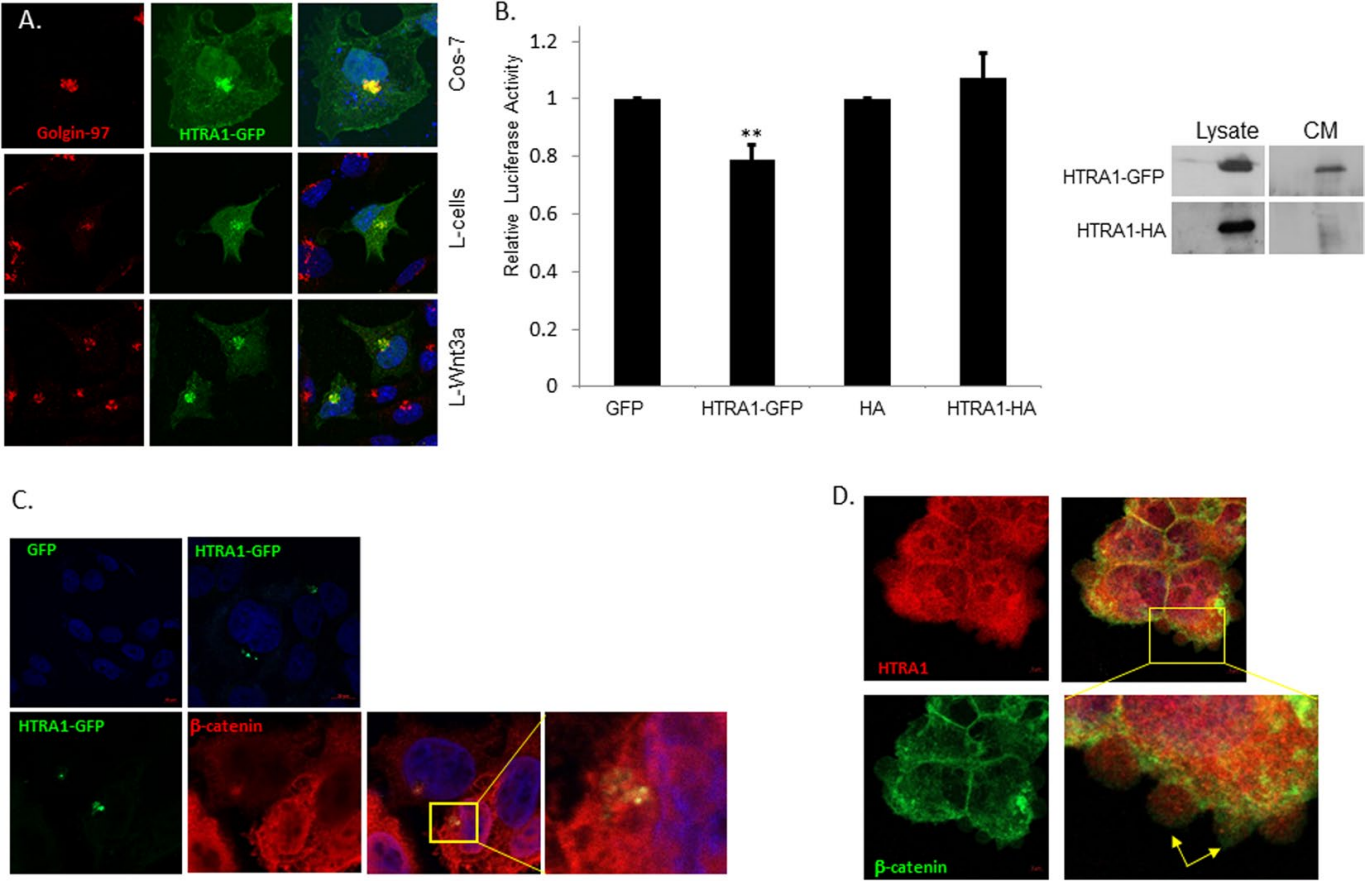
Secreted HTRA1 affects Wnt signaling. (**A**) COS7 cells, L cells, and L-Wnt3a cells were transfected with HTRA1-GFP (1 μg). Forty-eight hours after transfection, cells were fixed, permeabilized, and stained with an anti-golgin97 antibody. Alexa-546 fluorescent antibody was used and cell nuclei were then stained with DAPI. HTRA1 was detected through the GFP tag. Cells were visualized using confocal microscopy. (**B**) HEK293T cells were transfected with the indicated plasmids and their respective controls and incubated for 24 h. Cells were harvested and CM was collected for Western blot analysis (right panel). SW480 cells were transfected with the pTOPFLASH and pFOPFLASH constructs as before. Twenty-four hours post-transfection, their medium was replaced with the indicated CM. Twenty-four hours later, the cells were harvested and subjected to a luciferase reporter assay (left panel). (**C**) HEK293T cells were transfected with the GFP or HTRA1-GFP constructs and CM was collected after 48 h. SW480 cells were incubated with the indicated CM for 24 h and IF assay was performed as described before. In the lower panel β-catenin was detected using the mouse anti-β-catenin (BD) specific antibody. (**D**) HEK293T cells were immunostained as described earlier with the rabbit anti-HTRA1 (ACRIS) and the mouse anti-β-catenin (BD) specific antibodies. Cells were visualized using confocal microscopy.

HTRA1 is a secreted protein^26,27^. Indeed, ectopic expressed HTRA1-GFP co-localizes with the Golgi marker Golgin-97 in different cell lines, which indicates that the protein enters the secretory pathway (Fig. 2A). Secreted HTRA1-GFP markedly decreased Wnt signaling, though to a lesser degree than the overexpressed protein (20% versus 50%, Figs 1C and 2B respectively). Interestingly, the HTRA1-HA protein did not enter the secretory pathway (not shown), could not be detected in CM (Fig. 2B, right panel) and subsequently, did not affect the Wnt pathway (Fig. 2B, left panel). The ability of secreted HTRA1 to affect signaling in recipient cells suggests that the protein may be internalized. Indeed, HTRA1-GFP from HEK293T cells overexpressing this plasmid was detected in SW480 treated cells (Fig. 2C, upper panel). Partial co-localization with β-catenin was observed in some cells (Fig. 2C, lower panel). The β-catenin protein was previously found in secreted exosomes^28^. Staining of HTRA1 revealed that the latter is also detected in “bleb”-like protrusions (arrows) that are generally associated with the secretion pathway (Fig. 2D).

### Catalytically inactive HTRA1 affects the Wnt signaling pathway

**Figure 3:**
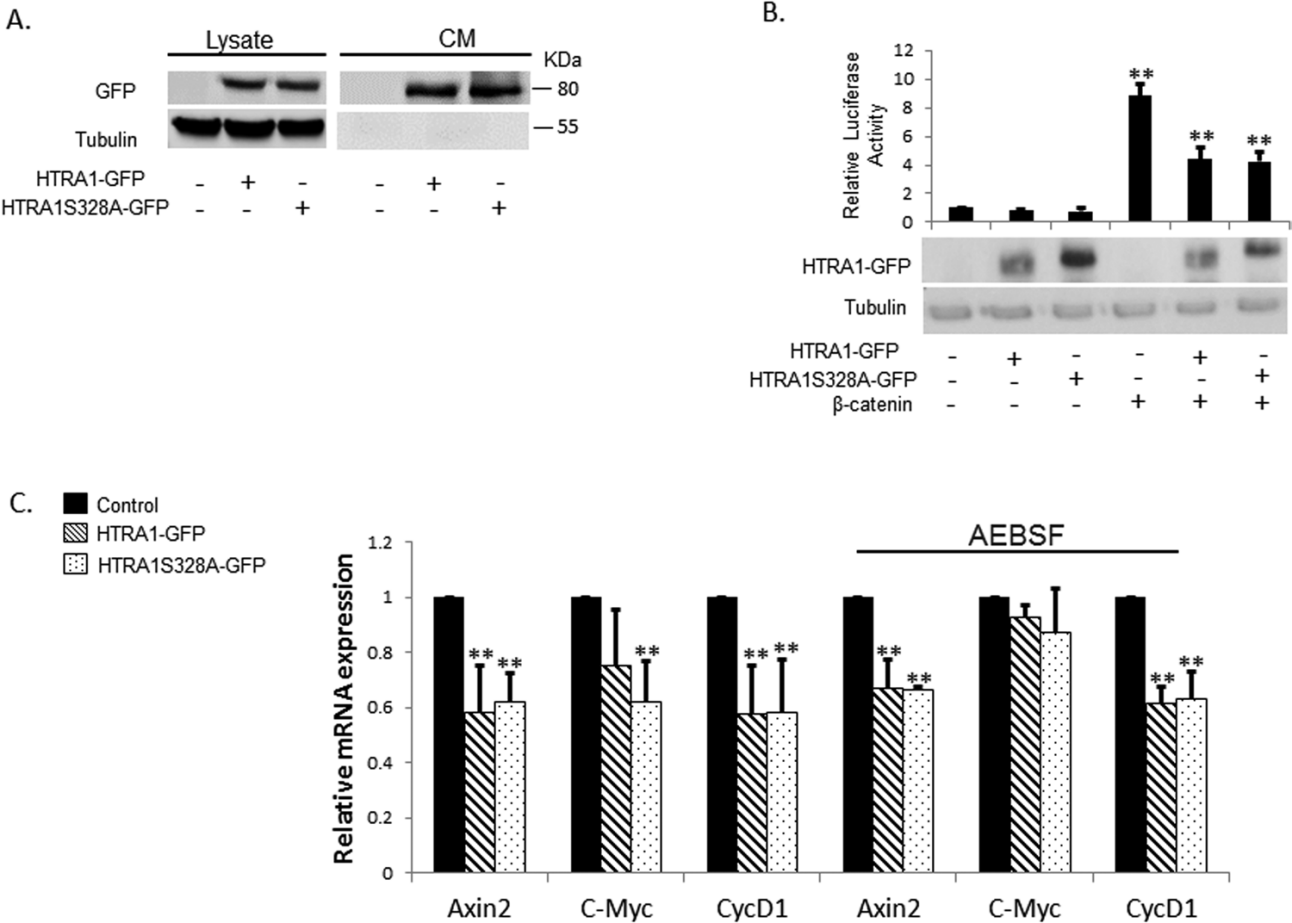
Catalytically inactive HTRA1 affects Wnt signaling. (**A**) HEK293T cells were transfected with HTRA1-GFP, HTRA1 S328A-GFP, or an empty pEGFP vector as a basal control (2 μg). Forty-eight hours after transfection, CM was collected and the cells were harvested. Both the medium and the lysates were subjected to Western blot analysis, using anti-GFP or anti-tubulin antibodies. (**B**) HEK293T cells were transfected with the pTOPFLASH/pFOPFLASH constructs (0.5 μg) and β-gal plasmid (0.1 μg), along with HTRA1-GFP, HTRA1 S328A-GFP, GFP-β-catenin, or an empty pEGFP vector as a basal control (1 μg). Forty-eight hours after transfection, cells were harvested and subjected to a luciferase assay or Western blot analysis, using anti-HA, anti-HTRA1, or anti-tubulin antibodies. (**C**) HEK293T cells were transfected with HTRA1-GFP, HTRA1 S328A-GFP, or an empty pEGFP vector as a basal control (2 μg). Forty-eight hours after transfection, cells were incubated with 50 μM serine protease inhibitor 4-(2-Aminoethyl) benzenesulfonyl fluoride hydrochloride (AEBSF) or dH_2_O as a control for 30 minutes at 37 °C. Cells were harvested and total RNA was extracted and converted into a more stable cDNA. The cDNA was subjected to real-time PCR assay as described earlier. (*p value < 0.05 **p value < 0.01, Student’s t-test).

HTRA1 contains a trypsin-like catalytic domain consisting of a crucial serine 328 residue, which when mutated, causes the protein to lose its enzymatic activity^27^. We used this mutant form to further decipher the mechanism by which HTRA1 affects the Wnt pathway. First, we demonstrate that both the wild-type and mutant forms of HTRA1 are properly expressed and secreted (Fig. 3A). Next, we co-transfected HEK293T cells expressing the β-catenin protein with either the wild-type or mutated HTRA1 along with the reporter vectors. Our results indicate that HTRA1 can inhibit the Wnt cascade regardless of its enzymatic activity (Fig. 3B). We then examined how both the wild-type and mutated HTRA1 forms affect the regulation of the Wnt target genes c-myc, axin-2, and cyclinD1. HEK293T cells were transfected with HTRA1-GFP, HTRA1-S328A-GFP, or the GFP empty vector. Finally, the cells were harvested and RNA was extracted. The qRT-PCR assay was performed on the indicated genes and β-actin served as an internal control. As can be seen from Fig. 3C, the catalytically inactive HTRA1 has a similar effect on the genes tested compared with the wild-type form. Moreover, the addition of AEBSF, a serine protease inhibitor, had no apparent effect on these genes levels, further supporting our hypothesis that the HTRA1 effect on Wnt signaling is independent of its catalytic activity. As expected, the ectopic expression of HTRA1 led to a significant decrease in the Wnt target genes Axin2 and cyclidD1, though the effect on C-myc is more subtle. We believe that the changes in C-Myc levels are most likely attributed to its promoter conformation or to the notion that C-Myc can be differentially expressed as compared with other Wnt target genes, depending on the cell cycle^29^, the chronological expression^30^, and the promoter conformation^31^.

### HTRA1 interacts with β-catenin

**Figure 4:**
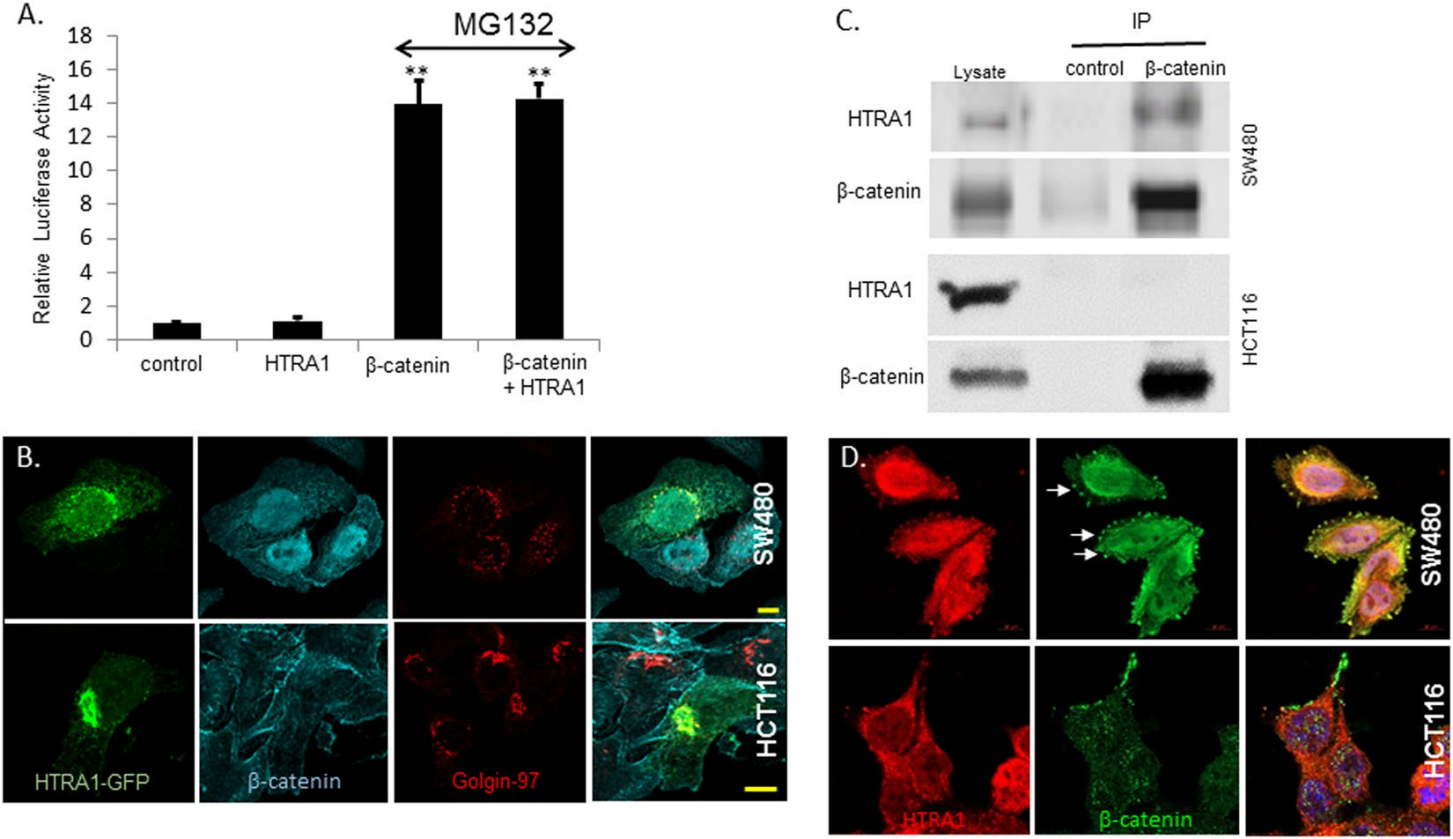
HTRA1 interacts with β-catenin. (**A**) HEK293T cells were transfected with the pTOPFLASH/pFOPFLASH constructs (0.5 μg) and β-gal plasmid (0.1 μg), along with HTRA1-HA, HA-β-catenin, or an empty HA vector as a basal control (1 μg). Forty-eight hours after transfection, cells were treated for five hours with either MG132 (25 µM) or DMSO as a control. The cells were harvested and subjected to a luciferase assay. (*p value < 0.05 **p value < 0.01, Student’s t-test). (**B**) SW480 and HCT116 cells were transfected with HTRA1-GFP (1 μg). Forty-eight hours after transfection, cells were fixed, permeabilized, and stained with an anti-active β-catenin (ABC) and anti-golgin97 antibodies. Alexa-546 and Alexa-647 (respectively) were used as fluorescent antibodies and HTRA1 was detected through the GFP tag. Cells were visualized using confocal microscopy. (**C**) SW480 or HCT116 cell lysates were subjected to co-immunoprecipitation using an anti-β-catenin antibody or a non-specific IgG control. Lysates and immunoprecipitants were analyzed by western blotting using anti-HTRA1 or anti-β-catenin antibodies. (**D**) SW480 and HCT116 cells were immunostained with the rabbit anti-HTRA1 (ABGENT) and the mouse anti-total-β-catenin (BD) specific antibodies, as described. Alexa-546 and Alexa-488 (respectively) were used as fluorescent secondary antibodies.

Our results suggest that when β-catenin cannot be phosphorylated, HTRA1 has no effect on the Wnt signal (Fig. 1A). Moreover, when the proteasome inhibitor MG132 was used, the activity of HTRA1 was abolished (Fig. 4A). However, in SW480 cells that express a wild-type β-catenin protein, HTRA1 downregulates the pathway, as shown by the overexpression and depletion experiments (Fig. 1C,D). Examining the subcellular localization of HTRA1-GFP in SW480 cells that express nuclear β-catenin and in HCT116 where β-catenin is mostly membrane bound reveal that HTRA1 partly localizes to the nucleus in SW480 cells, whereas in HCT116 cells HTRA1 remains in the Golgi (Fig. 4B). To further study the mechanism underlying the HTRA1′s inhibitory effect on the Wnt cascade, we tested the potential interactions between HTRA1 and β-catenin. In this experiment two cell types were used to perform immunoprecipitation and immunofluorescent assays (Fig. 4C and D respectively). SW480 cells express wild-type β-catenin, whereas HCT116 cell line expresses a mutated β-catenin carrying a 3-bp deletion that removes the amino acid Ser45. This residue is one of the residues phosphorylated by GSK3β and is frequently mutated in tumors^32^. According to our results, only the wild-type β-catenin binds the HTRA1 protein, as shown in Fig. 4C. Immunostaining of SW480 cells with a specific anti-HTRA1 antibody reveals some co-localization between the two proteins (Fig. 4D, arrows, upper panel). This co-localization was not detected in HCT116 cells (Fig. 4D lower panel).

### The effect of HTRA1 on Wnt signaling is mediated through its kazal and PDZ domains

**Figure 5:**
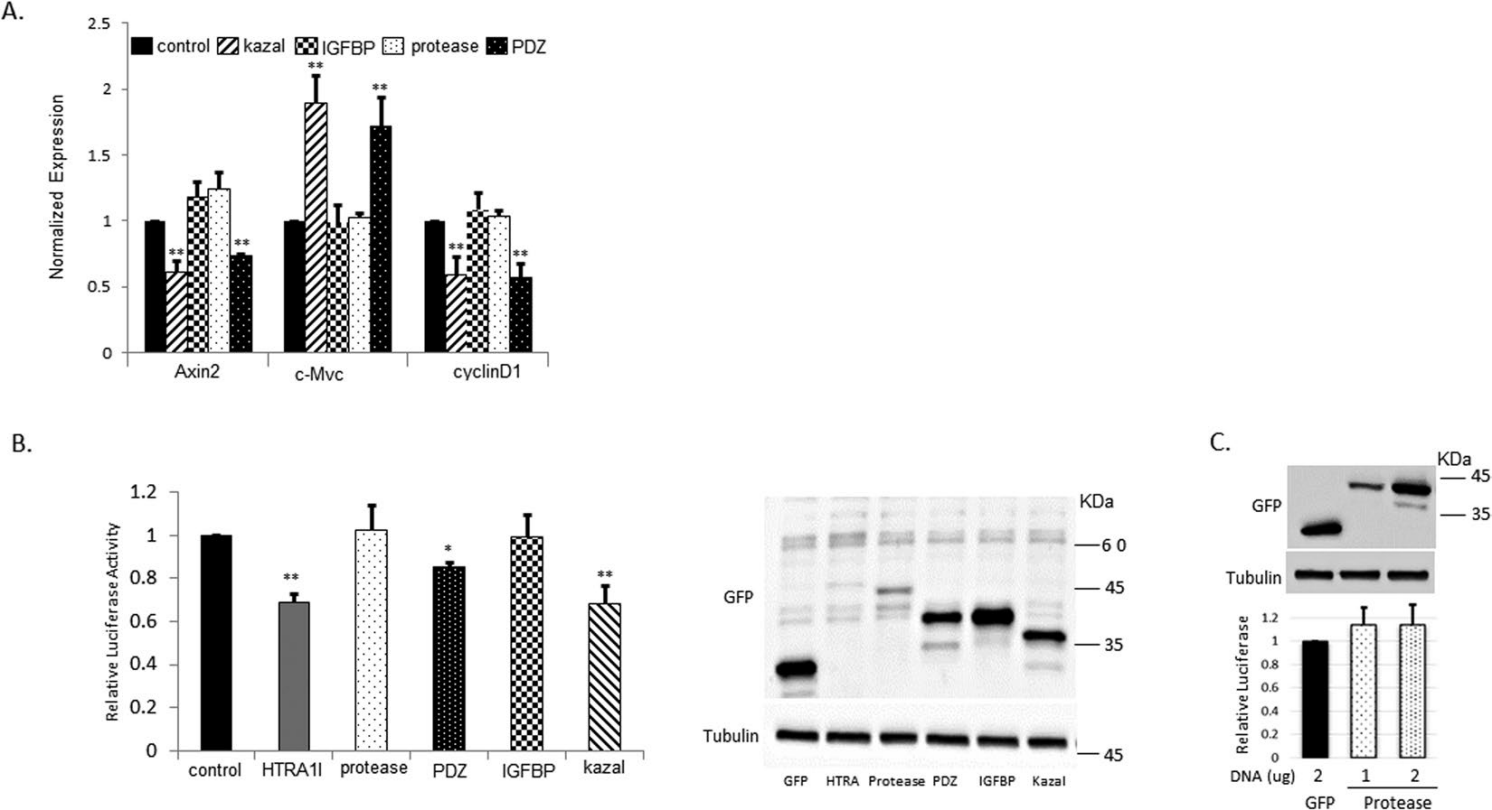
The effect of HTRA1 on Wnt signaling is mediated via its kazal and PDZ domains. (**A**) HEK293T cells were transfected with GFP-IGFBD, GFP-Kazal, GFP-protease, GFP-PDZ, or an empty pEGFP vector as a control (2 μg). Forty-eight hours after transfection, cells were harvested; total RNA was extracted from the cells and converted into a more stable cDNA. Quantitative real-time PCR was performed as described (*p value < 0.05 **p value < 0.01, Student’s t-test). (**B**) SW480 cells were co-transfected with HTRA1-GFP, GFP-IGFBD, GFP-Kazal, GFP-protease, GFP-PDZ, or an empty pEGFP vector as a control (1 μg) along with the pTOPFLASH/pFOPFLASH constructs (0.5 μg) and renilla plasmid (0.1 μg). Forty-eight hours after transfection, cells were harvested and subjected to a luciferase assay as described previously. Right panel depicts western blot analysis of the different HTRA1 domains (detected by anti-GFP antibody). (**C**) SW480 cells were co-transfected with two concentrations of the GFP-protease domain or an empty pEGFP vector as a control, along with the pTOPFLASH/pFOPFLASH constructs (0.5 μg) and renilla plasmid (0.1 μg). Forty-eight hours after transfection, cells were harvested and subjected to a luciferase assay as described previously. Upper panel depicts western blot analysis showing that the higher DNA concentration lead to an increased protein expression level (detected by anti-GFP antibody).

HTRA1 is composed of four distinct protein domains: an Insulin-like growth factor binding domain (IGFBD), a kazal-type motif (Kazal), a trypsin-like peptidase (proteolytic) domain, and a PDZ domain^27^. In an attempt to better understand the molecular mechanism that contributes to the HTRA1 effect on the Wnt pathway, we tested how each domain affects the expression levels of specific Wnt target genes (Fig. 5A). Briefly, HEK293T cells were transfected with the different domains and 48 hours later, cells were harvested; RNA was extracted and the expression levels of Axin, c-Myc, and Cyclin D1 were measured using qRT-PCR. The results show that the Kazal and PDZ domains affected the tested genes similarly to the effect of the full-length HTRA, except for their effect on C-myc. Those two domains reduced the levels of Axin2 and Cyclin D1, yet elevated the mRNA expression levels of C-myc. We then tested the effect of the different domains on Wnt signaling and found that indeed the PDZ and Kazal domains had a similar effect as the full length HTRA1. The protease and IGFPB show no significant effect on Wnt activation (Fig. 5B). The expression level of the different domains varies, however the effect on the Wnt cascade is not influenced by their amounts. To verify that result we tested two concentrations of the protease domain and show that even when expression levels are increased the Wnt signaling remains unaffected (Fig. 5C).

### The expression levels of the HTRA1 tumor suppressor are reduced in Wnt signaling-activated cells

**Figure 6:**
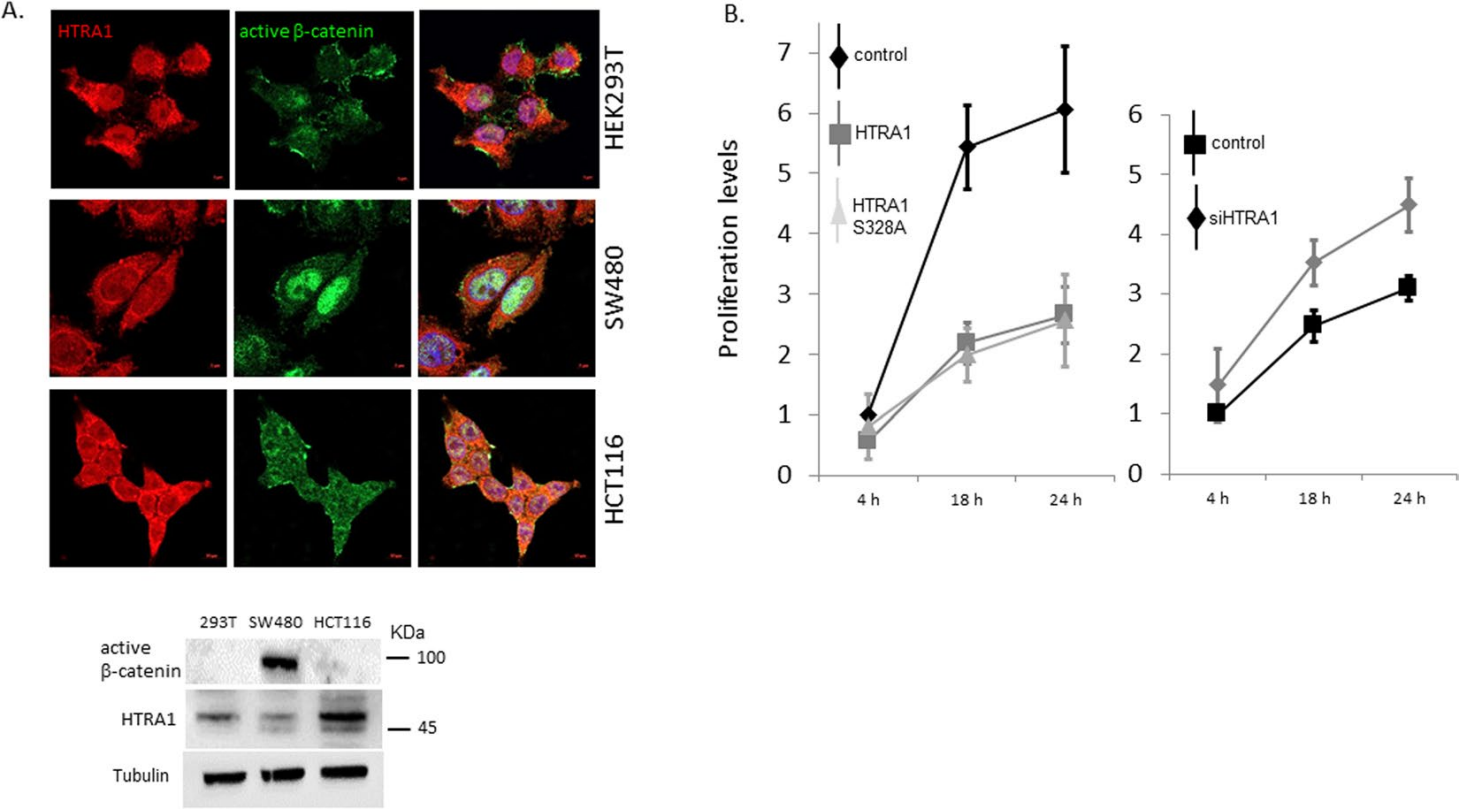
The expression levels of the HTRA1 tumor suppressor are decreased in Wnt signaling-activated cells. (**A**) HEK293T, SW480 and HCT116 cells were immunostained with an anti-HTRA1 and the anti-active ß-catenin specific antibodies as described. Cells were visualized using confocal microscopy. HEK293T, SW480 and HCT116 cells were harvested and their lysates were subjected to western blot analysis using anti-HTRA1, anti-active β-catenin, or anti-tubulin antibodies. (**B**) HEK293T cells were transfected with the indicated vectors (left panel) or with HTRA1 siRNA (100 nM) or scrambled siRNA as a control (right panel). Twenty-four hours following transfection, cells were split into a 96-well plate. Fourth-eight hours after transfection, cells were treated with XTT labeling mixture according to the XTT cell proliferation kit’s instructions. Cells were incubated at 37 °C, and the proliferation levels were measured using an ELISA reader at 450 nm, with a reference wavelength at 650 nm, every 4 h, 18 h, and 24 h. The results indicate the relative absorbance values normalized by the reference wavelength. Each bar represents the mean ± SD of three independent experiments (performed in triplicate).

Numerous studies have linked HTRA1 to tumorigenesis since it has been found to be down-regulated in many tumors including prostate cancer, ovarian cancer, and melanoma. We examined the expression pattern of HTRA1 in HEK293T cells and in the CRC cell lines SW480 and HCT116. Immunostaining of endogenous HTRA1 in these cells revealed a slight reduction in HTRA1 levels in the SW480 cells (Fig. 6A; upper panel). This result was further strengthen by a western blot analysis showing that endogenous HTRA1 levels are reduced in the CRC cell line SW480 compared with the non-cancerous original HEK293T cells (Fig. 6A; lower panel). In order to better understand the effect of the HTRA1 protein on the oncogenic traits, we examined the proliferation levels of HEK293T cells overexpressing HTRA1-GFP, HTRA1-S328A-GFP, or an empty pEGFP vector as a control. The proliferation levels were examined using the XTT cell proliferation assay 4, 18, or 24 hours post treatment with an XTT labeling mixture. As expected, cells overexpressing HTRA1 (the wild-type or mutated form) show a decrease of about 60% in their proliferation abilities (Fig. 6B left panel). Again, HTRA1 S328A exhibited an effect similar to the wild-type form, supporting our assumption that the effect of HTRA1 on Wnt signaling is independent of its catalytic activity. We also examined the effect of HTRA1 silencing on the proliferation abilities of HEK293T cells transfected with HTRA1 siRNA compared with a control (Fig. 6B right panel). HTRA1 depletion resulted in an increase of about 15% of the proliferation abilities of the cells.

### The activity of HTRA1 may be dependent on its tertiary structure

**Figure 7:**
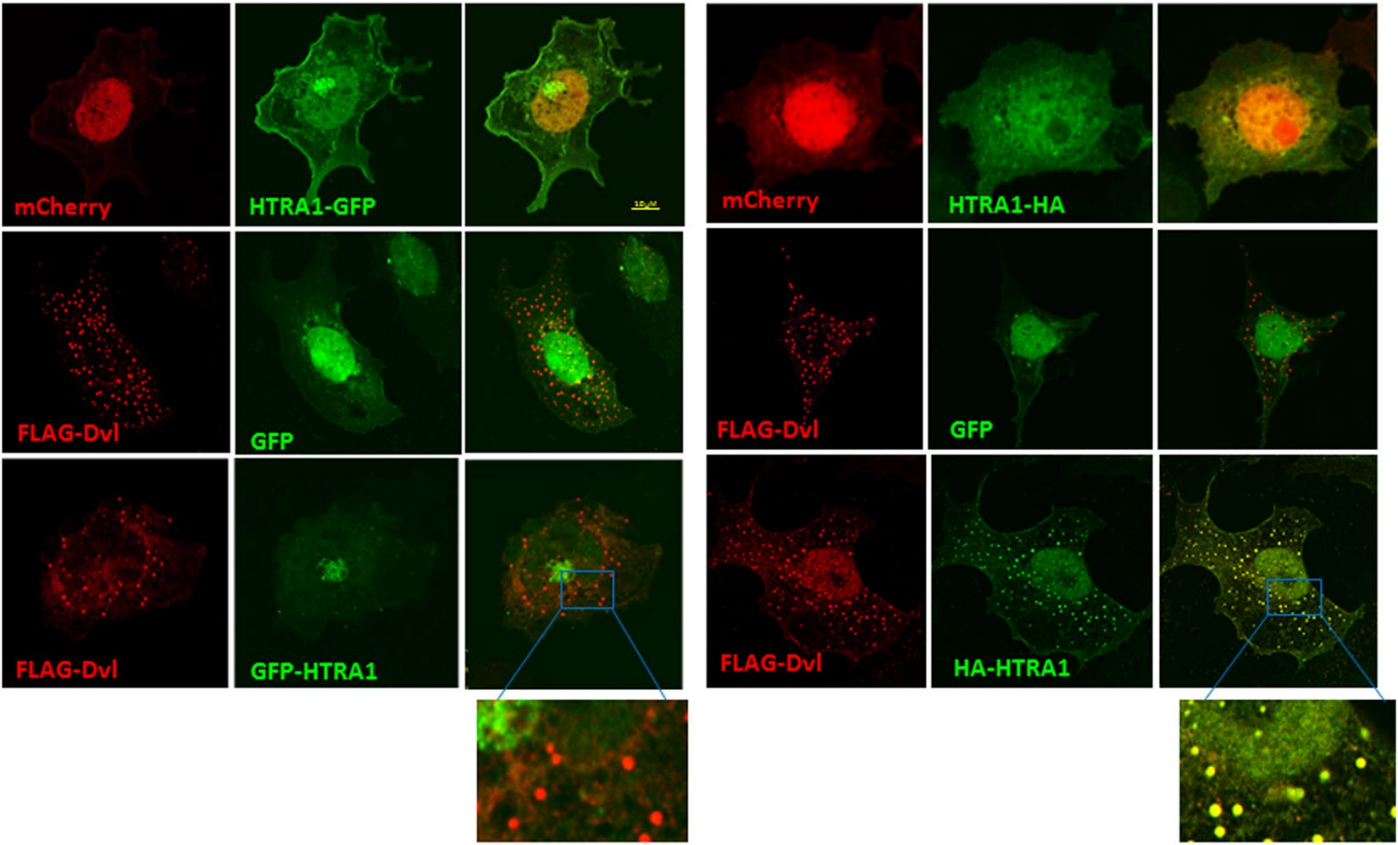
The activity of HTRA1 may be dependent on its tertiary structure. COS7 cells were transfected with GFP, HTRA1-GFP, or HTRA1-HA, along with mCherry or FLAG-dvl (1 μg). Forty-eight hours after transfection, cells were fixed, permeabilized, and stained with an anti-FLAG or anti-HA antibody. Alexa-546 and Alexa-488 were used as fluorescent antibodies, GFP and HTRA1-GFP were detected through the GFP tag, and mCherrry was detected through the cherry tag. Cells were visualized using confocal microscopy.

HTRA proteins have a complex structural organization that enables their increased involvement in numerous cellular processes. HTRA2, for example, is arranged in a way that masks its active site with a flexible linker at the PDZ-protease interface. A recent study suggests that the relative orientations and crosstalk between the different HTRA2 domains are actually crucial for defining its functions^33^. In the course of the current study we discovered some significant differences in the cellular distribution and secretion between the GFP and the HA-tagged HTRA1 constructs. As shown in Fig. 7, the expression pattern of HTRA1-HA is diffused throughout the cell, with no distinct Golgi staining that is very clear, as in the case of the HTRA1-GFP construct. Furthermore, when Disheveled (a Wnt pathway component that also contains a PDZ domain) was co-transfected with HTRA1-HA, the two proteins co-localized to puncta-like structures that are a familiar trait of disheveled overexpression. Such co-localization was not detected when HTRA1-GFP was overexpressed. Additionally, the secretion of HTRA1-HA is lower than that of HTRA1-GFP (Fig. 2B), even though their expression levels are similar. As we showed earlier (Fig. 1C), the effect of the two constructs on Wnt signaling in SW480 cells is also different. HTRA1-HA exhibits a lesser inhibitory effect compared with the HTRA1-GFP overexpression. Since our results indicate that the PDZ and kazal domains might be involved in HTRA1’s function on reducing the Wnt cascade, we hypothesized that the different tags induce conformational changes that result in a different tertiary structure and consequently, differences in sequestering and activity.

## Discussion

The HTRA1 protein is a member of the human HTRA family of serine proteases, which are found in a variety of species, including bacteria, plants, and mammals^34^. HTRA1 is conserved and is involved in numerous biological processes indicating its importance in human physiology. Previously It has been shown that HTRA1 is down-regulated in cancer of diverse origins, such as medulloblastoma^18^, prostate cancer, malignant melanoma^20^, metastatic sarcoma, lung carcinoma^21^, mesothelioma^22^, and ovarian cancer^18^. In breast cancer, lower HTRA1 expression levels were found to correlate with poor prognosis^23^. HTRA1 down-regulation is highly important in cancer progression and in acquiring aggressive tumor traits, mainly due to the elimination of its tumors pro-apoptotic and anti-growth properties. In the current study, we show that, in accordance with its tumor suppressive activity, HTRA1 acts as an inhibitor of the canonical Wnt cascade by down-regulating the Wnt signal and the expression of specific Wnt target genes. We showed that overexpression of HTRA1 in Wnt-activated cells reduces both TCF/β-catenin-mediated activity and β-catenin expression levels. In addition, we demonstrated that overexpression of HTRA1 altered the mRNA expression of several Wnt target genes, including Axin-2, c-myc, and cyclinD1. A number of studies have discussed the differences in the expression of Wnt target genes and have shown that c-myc can be differentially expressed as compared with other Wnt target genes that are dependent on the cell cycle^29^, chronological expression^30^, and whose expression levels are dependent on promoter conformation^31^. Interestingly, whereas the ectopic expression of HTRA1 led to a significant decrease in the Wnt target genes Axin2 and cyclidD1, the effect on C-myc was much more subtle and occasionally (not shown) even reversed. We speculate that the changes in the levels of C-myc may be attributed to its promoter conformation, as was recently shown; (detailed model is presented in a recent study^31^). This model states that conformational changes in the promoter region may influence C-myc expression in CRC cells. It is possible that under certain conditions, the β-catenin/HTRA1 complex harbors high affinity to remote Wnt responsive elements (WRE), thus enhancing chromatin changes that specifically alter C-myc expression. There is emerging data suggesting that the Wnt and TGF-β pathways crosstalk in different ways. For example, it was proposed, that along the colonic crypt–villus axis, proliferation, migration, differentiation and compartmentalization are controlled by TGF-β- and Wnt gradients. The presence of TGF-β signaling and the absence of Wnt signaling in the villus compartment result in rapid cell cycle arrest and differentiation^35^. Thus, these pathways may control the switch between proliferation and differentiation. The relationship between the pathways is context dependent and is evident in different cell types, both in normal development and in disease^35–37^. Interestingly, it has been shown that the activity of HTRA1, which is impaired in cerebral autosomal recessive arteriopathy with subcortical infarcts and leukoencephalopathy (CARASIL) patients, affects TGF-β signaling^38–40^. Importantly, using embryonic fibroblasts from HTRA1-knockout mice, it has been shown that HTRA1 antagonizes TGF-β signaling by cleaving its receptors to affect bone mass^41^ and has a role in angiogenesis via TGF-β signaling^42^. Our data indicates that TGF-β may overturn the effect of HTRA1 on Wnt target genes expression suggesting that the connection between HTRA1 and the Wnt pathway is governed by TGF-β. These findings should be further examined by using fibroblasts obtained from HTRA1 null mice.

Next we demonstrated that silencing the expression of the endogenous HTRA1 resulted in increased Wnt signaling. It also led to elevation of all Wnt target genes that were tested: Axin2, C-myc, and cyclin D1. This strongly supports our assumption that HTRA1 functions as a novel repressor of the canonical Wnt pathway. Note that silencing of the HTRA1 gene has been previously linked to colorectal cancer, in a yet unexplained mechanism, and to numerous cancerous phenotypes such as increased proliferation, DNA instability, and failure in DNA damage checking^25^. HTRA1 is a secreted protein with a widespread pattern of expression in the human body, and with modulated levels of expression according to the different activities of the tissues^43^. We tested the ability of secreted HTRA1 to suppress Wnt signaling by incubating cells with HTRA1-cultured media and by examining the TCF/β-catenin-mediated transcription. We show that GFP tagged-HTRA1 protein is secreted to the media and then can be internalized by recipient cells and affect the Wnt signal, although the effect is weaker than that of the overexpressed protein. HTRA1 is composed of four distinct domains: an Insulin-like growth factor binding domain (IGF-BD), a kazal-type motif (KM), a trypsin-like peptidase (proteolytic) domain, and a PDZ domain. The trypsin-like catalytic domain of the protein is responsible for its enzymatic activity and the serine at position 328 is crucial for its catalytic activity. We have used a catalytically inactive HTRA1 form (S328A)^44^ to examine its effect on Wnt signaling and on the transcription levels of several Wnt target genes. Importantly, we found that both the wild-type and the mutated forms of HTRA1 reduced the canonical Wnt signal levels to the same extent and similarly affected the expression of the Wnt target genes. Moreover, the effect of both proteins on different Wnt target genes in the presence of a serine protease inhibitor is also similar. These results indicate that HTRA1’s effect on Wnt signaling is independent of its catalytic activity, and that it is probably mediated through a different mechanism that remains to be fully understood. When the effect of each of HTRA1’s domains was examined separately, two domains exhibited behavior similar to that of the full-length protein, the Kazal domain, and the PDZ domain. Interestingly, both domains also physically interact with the β-catenin protein (not shown). This might indicate that the effect of the protein is mediated through one or both of the domains. HTRA proteins are known for their complex structural organization, which enables them to mediate a wide array of biological functions^33^. Although debatable, some studies claim that HTRA1 can act as a chaperone or that it harbors chaperone-like activity. In this work, we showed that the effect of HTRA1 on the Wnt signal is mediated through its interaction with the β-catenin protein. We showed that cells expressing high levels of endogenous HTRA1 exhibit lower levels of active β-catenin and vice versa. Additionally, we showed that HTRA1 and β-catenin physically interact. These findings may indicate that the effect of HTRA1 is mediated through a physical interaction with β-catenin, which results in reduced activity. In addition, we have shown that when β-catenin is mutated in a form that cannot be phosphorylated and degraded by the proteasome, HTRA1 has no effect on the Wnt signal and its ability to interact with β-catenin is abolished. Similarly, the proteasome inhibitor MG132 also ablated HTRA1’s capacity to inhibit Wnt signaling. These findings imply that β-catenin phosphorylation is crucial for HTRA1’s influence on the Wnt cascade. In order to better understand why HTRA1 is often implicated as a tumor suppressor, we examined its effect on cellular proliferation abilities, and as an indicator of the tumorigenic traits of cells. We found that overexpressed HTRA1 (both the wild type and the catalytically inactive protein) reduces the proliferation levels of the cells, whereas endogenous HTRA1 silencing increases cellular proliferation. This evidence supports HTRA1’s role as a tumor suppressor. Interestingly, the HA-tagged HTRA1 protein does not accumulate in the Golgi and is not secreted. A previous study showed that tags, and specifically an HA tag, can change the conformation and activity of a protein specifically in cases where protein function is based on conformational changes^45^. Indeed, HTRA proteins possess a complex structural organization, which enables them to fulfill a wide array of cellular functions^33^. Interestingly, the HA-tagged HTRA1 protein is strongly recruited to the Disheveled signalosomes. Dvl is a known activator of the Wnt cascade and is often found in puncta-like structures when overexpressed^46^. Interestingly, it was recently shown that Dvl utilizes conformational changes to regulate the Wnt signaling pathways. For example, a C-terminal motif can intrinsically bind the Dvl PDZ domain in order to form a “closed” conformation^47^. It is possible that the GFP tag mimics an interaction/modification that keeps HTRA in a conformation programmed for secretion, whereas the HA tag exposes its PDZ domain to be captured in the signalosomes by the DVl C-terminal motif. The assembly of those signalosomes that are important for the transduction of the Wnt pathway may lead to loss of HTRA1 activity. As the Wnt signaling pathway plays important roles in both normal development and pathogenesis, these findings may provide new molecular clues to the way this important pathway transduces its signal. Since the pathway is so complex and highly controlled, new genes, proteins, and other regulatory components are still frequently being identified. Characterizing these new components may shed new light on molecular events that lead to cancer development and progression and thus may hold therapeutic potential for its treatment.
